# *N*′-[(*E*)-5-Oxopyrrolidin-2-yl­idene]pyridine-2-carbohydrazide

**DOI:** 10.1107/S241431462500896X

**Published:** 2025-10-28

**Authors:** Yurii S. Bibik, Hanna V. Ivanova, Dmytro M. Khomenko, Roman O. Doroshchuk, Alexandru-Constantin Stoica

**Affiliations:** a Department of Chemistry Kyiv National Taras Shevchenko University Hetman Pavlo Skoropadskyi Street 12 Kyiv 01033 Ukraine; b Enamine Ltd, Winston Churchil st. 78, Kyiv 02094, Ukraine; c ChemBioCenter Kyiv National Taras Shevchenko University Hetman Pavlo Skoropadskyi Street 12 Kyiv 01601 Ukraine; d ‘Petru Poni’, Institute of Macromolecular Chemistry, Aleea Grigore Ghica Vodă 41A, Iaşi 700487, Romania; University of Aberdeen, United Kingdom

**Keywords:** crystal structure, X-ray crystallography, pyridine-2-carbohydrazide

## Abstract

In the title compound, C_10_H_10_N_4_O_2_, the dihedral angle between the planes of the pyridine and oxo­pyrroli­dine rings is 6.9 (2)°. In the crystal, inversion dimers linked by pairwise N—H⋯O hydrogen bonds generate *R*_2_^2^(14) loops.

## Abstract 2

In the title compound, the dihedral angle between the planes of the pyridine and oxo­pyrroli­dine rings is 6.9 (2)°. In the crystal, inversion dimers linked by pairwise N—H⋯O hydrogen bonds generate *R*_2_^2^(14) loops.

## Graphical abstract

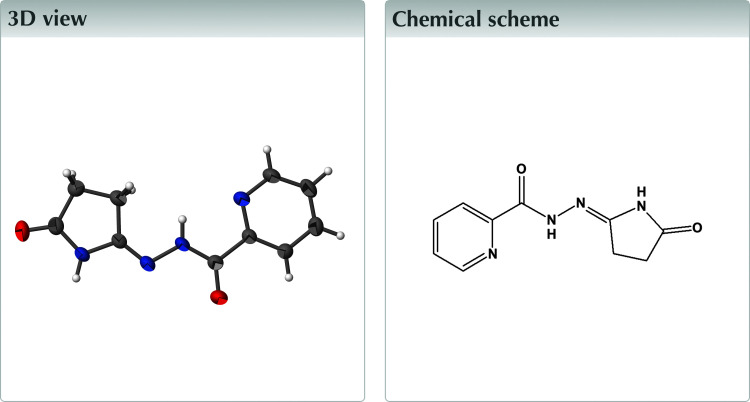

## Structure description

Pyridine-2-carbohydrazides are organic compounds containing a pyridine ring substituted with a carbohydrazide (–CONHNH_2_–) group at the 2-position. These compounds are valuable in organic and medicinal chemistry due to their diverse biological activities and reactivity. They have been studied extensively for their medicinal properties (*e.g.* Khan *et al.*, 2022 ▸; Pitucha *et al.*, 2020 ▸; Marinescu & Popa, 2022 ▸). As part of our work in this area, we now report the synthesis and structure of *N*′-[(*E*)-5-oxopyrrolidin-2-yl­idene]pyridine-2-carbohydrazide.

**Figure 1:**
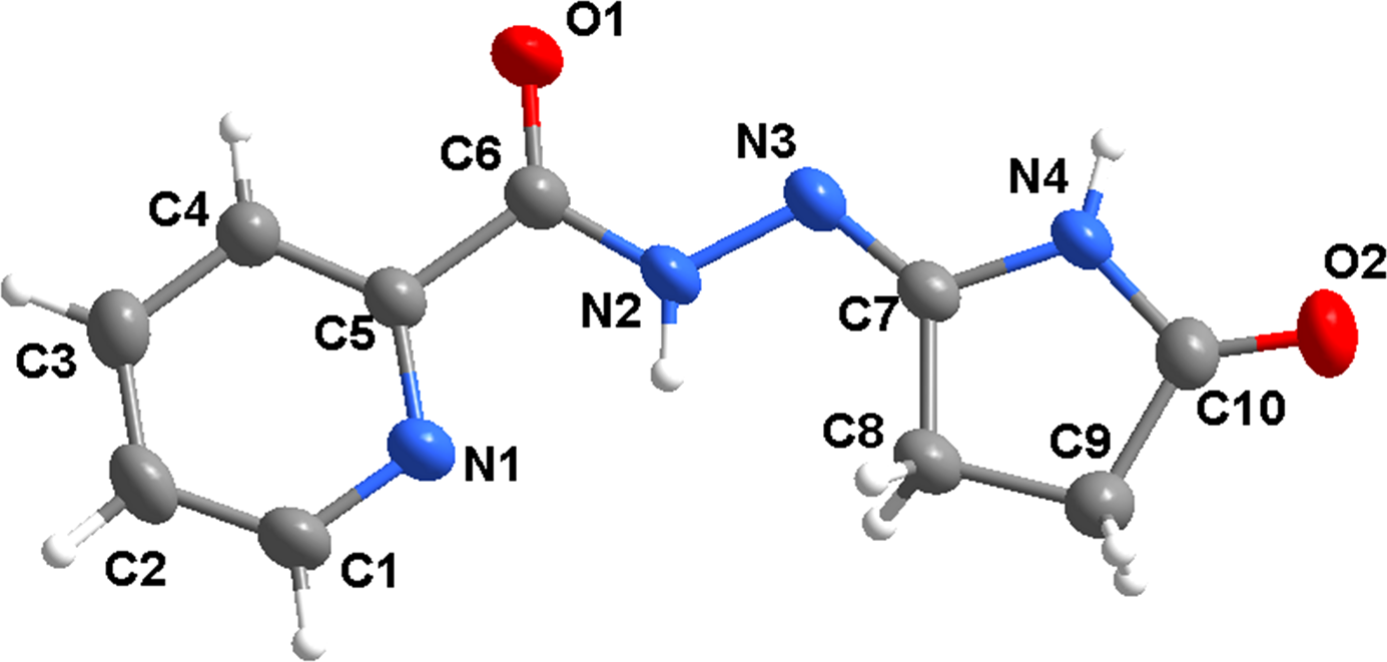
The mol­ecular structure of the title compound, with displacement ellipsoids drawn at the 50% probability level.

The title compound crystallizes in the triclinic space group *P*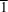 with one mol­ecule in the asymmetric unit (Fig. 1 ▸). The mol­ecule is not exactly planar, but can be divided into three almost planar fragments, *viz.* a pyridine ring, a carbohydrazide unit (O1/C6/N2/N3) and an oxopyrrolidine ring. The carbohydrazide unit forms dihedral angles of 3.9 (2) and 5.2 (2)° with the N1/C1–C5 pyridine and N4/C7–C10 oxopyrrolidine rings, respectively. The dihedral angle between the planes of the pyridine and oxopyrrolidine rings is 6.9 (2)°. Pyridine atom N1 and carbohydrazide atom N2 are *cis* with respect to each other [torsion angles N1—C5—C6—N2 and N1—C5—C6—O1 = −0.9 (3) and 177.6 (2)°, respectively]. Hydrazide atom H2 and oxopyrrolidine atom H4 are *trans* with respect to each other [torsion angle N2—N3—C7—N4 = 179.33 (18)°]. In addition, the C6—N2 bond length of 1.322 (3) Å agrees well with equivalent bonds in similar structures, being inter­mediate between a typical C—N single bond (∼1.47 Å) and a C=N double bond (∼1.29 Å). The N2—N3 bond length of 1.396 (2) Å also shows partial double-bond character, suggesting extensive delocalization in the compound (Singh *et al.*, 2006 ▸).

**Figure 2:**
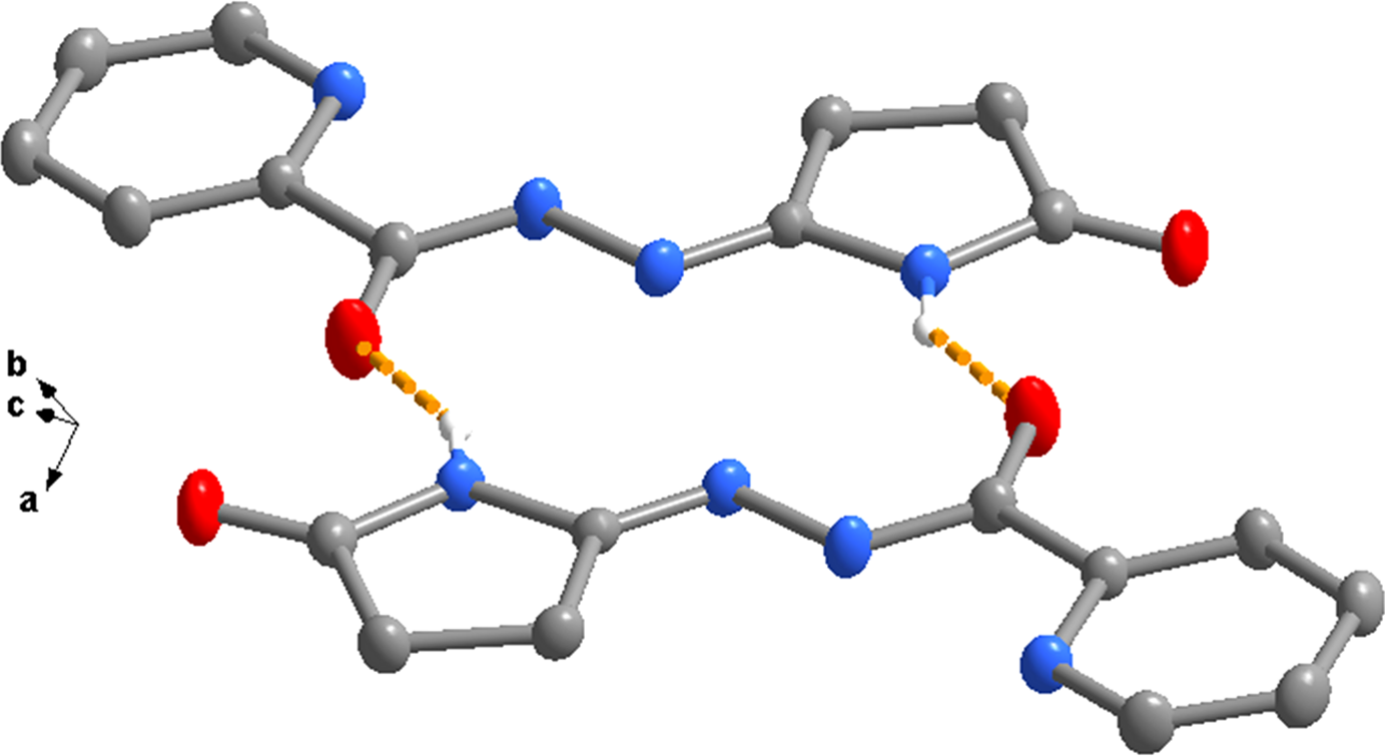
A dimer formed through pairwise N—H⋯O hydrogen bonds.

**Figure 3:**
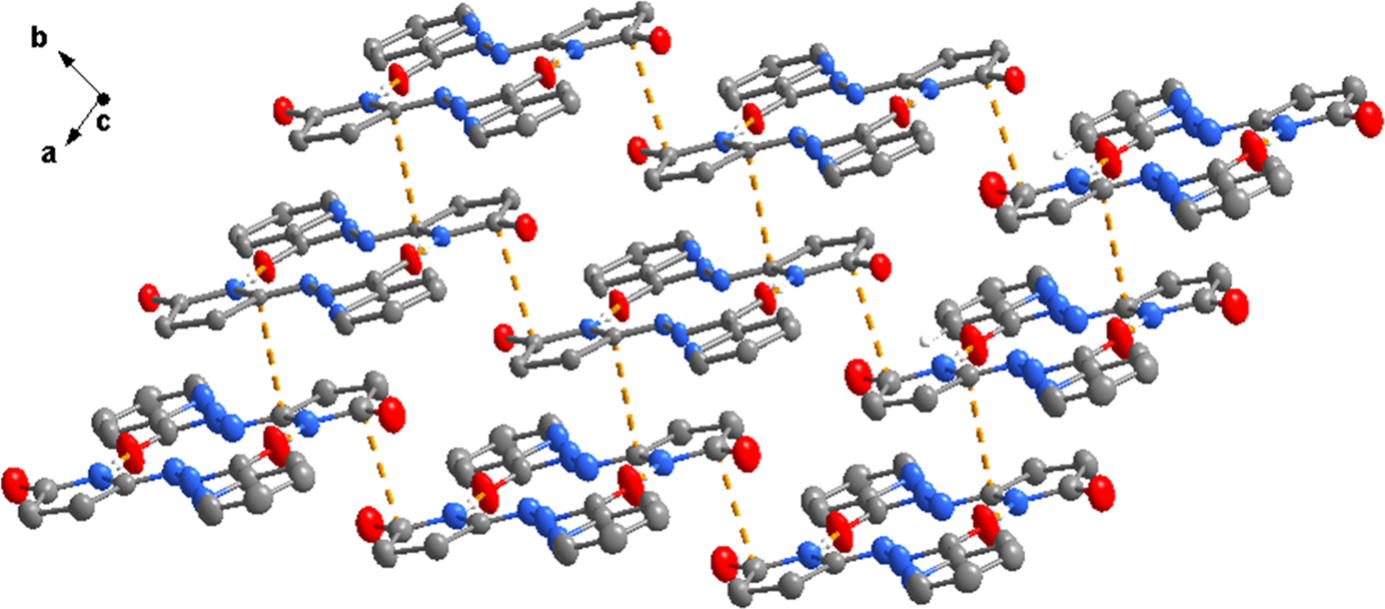
A view normal to the *ab* plane of the crystal structure of the title compound, showing the two-dimensional supra­molecular network.

In the extended structure, the mol­ecules forms dimers through pairwise N—H⋯O hydrogen bonds (Table 1 ▸ and Fig. 2 ▸), which generate 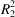
(14) loops. These dimers are further connected into a two-dimensional framework *via* short C⋯C contacts (3.23–3.39 Å), which are likely of van der Waals nature and occur between symmetry-related C atoms of adjacent mol­ecules (Fig. 3 ▸).

## Synthesis and crystallization

**Figure 4:**
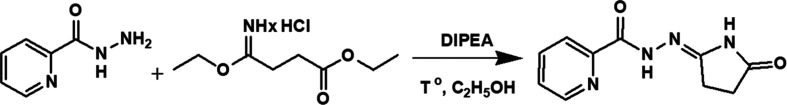
Synthesis scheme for the title compound.

A solution of pyridine-2-carbohydrazide in ethanol (2.05 g, 1.5 mmol in 125 ml) was added to a mixture of ethyl 4-eth­oxy-4-imino­butano­ate hydro­chloride (3.46 g, 1.65 mmol) and DIPEA (*N*,*N*-diiso­propyl­ethyl­amine) (2.85 ml, 1.72 mmol) in ethanol (125 ml) (Fig. 4 ▸). The reaction mixture was then refluxed for 8 h. After cooling to room temperature, the solvent was removed under reduced pressure. The resulting suspension was diluted with water (250 ml) and stirred to yield a white solid. The solid was collected by filtration, dried and recrystallized from aceto­nitrile solution. Crystals suitable for X-ray analysis were obtained by recrystallization from di­methyl­formamide (DMF) solution (yield: 8%, 0.3 g). ^1^H NMR (400 MHz, DMSO-*d*_6_): δ 11.31 (*s*, 0.5H), 10.96 (*s*, 0.5H), 10.74 (*s*, 0.5H), 10.61 (*s*, 0.5H), 8.66 (*d*, *J* = 4.6 Hz, 1H), 8.06–7.98 (*m*, 2H), 7.61 (*dd*, *J* = 3.8, 1.9 Hz, 1H), 2.92 (*t*, *J* = 7.2 Hz, 1H), 2.78 (*t*, *J* = 7.6 Hz, 1H). IR (KBr, ν, cm^−1^): 3443, 3315, 3112, 1759, 1681, 1655, 1592, 1538, 1432, 1227, 900, 818, 579, 428. LC/MS (ESI): *m*/*z* 219 [*M*H]^+^. Elemental analysis calculated (%) for C_10_H_10_N_4_O_2_: C 55.04, H 3.62, N 14.66; found: C 55.02, H 3.59, N 14.63.

## Refinement

Crystal data, data collection and structure refinement details are summarized in Table 2 ▸.

## Supplementary Material

Crystal structure: contains datablock(s) I. DOI: 10.1107/S241431462500896X/hb4539sup1.cif

Structure factors: contains datablock(s) I. DOI: 10.1107/S241431462500896X/hb4539Isup2.hkl

Supporting information containing IR and 1H-NMR spectra of the title compound. DOI: 10.1107/S241431462500896X/hb4539sup3.pdf

CCDC reference: 2495443

Additional supporting information:  crystallographic information; 3D view; checkCIF report

## Figures and Tables

**Table 1:** Hydrogen-bond geometry (Å, °)

*D*—H⋯*A*	*D*—H	H⋯*A*	*D*⋯*A*	*D*—H⋯*A*
N4—H4⋯O1^i^	0.86	2.02	2.822 (2)	154

Symmetry code: (i) 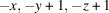.

**Table 2:** Experimental details

Crystal data	
Chemical formula	C_10_H_10_N_4_O_2_
*M*_r_	218.22
Crystal system, space group	Triclinic, *P* 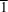
Temperature (K)	293
*a*, *b*, *c* (Å)	5.6332 (5), 7.6439 (7), 11.7293 (11)
α, β, γ (°)	95.859 (8), 96.353 (8), 99.682 (8)
*V* (Å^3^)	491.04 (8)
*Z*	2
Radiation type	Mo *K*α
μ (mm^−1^)	0.11
Crystal size (mm)	0.18 × 0.12 × 0.04

Data collection	
Diffractometer	Rigaku Xcalibur Eos
Absorption correction	Multi-scan (*CrysAlis PRO*; Rigaku OD, 2021 ▸)
*T*_min_, *T*_max_	0.716, 1.000
No. of measured, independent and observed [*I* > 2σ(*I*)] reflections	4130, 1734, 1120
*R*_int_	0.037
(sin θ/λ)_max_ (Å^−1^)	0.595

Refinement	
*R*[*F*^2^ > 2σ(*F*^2^)], *wR*(*F*^2^), *S*	0.049, 0.121, 1.01
No. of reflections	1734
No. of parameters	146
H-atom treatment	H-atom parameters constrained
Δρ_max_, Δρ_min_ (e Å^−3^)	0.17, −0.17

Computer programs: *CrysAlis PRO* (Rigaku OD, 2021 ▸), *SHELXT2018* (Sheldrick, 2015*a* ▸), *SHELXL2018* (Sheldrick, 2015*b* ▸) and *OLEX2* (Dolomanov *et al.*, 2009 ▸).

## full crystallographic data

### N'-[(E)-5-Oxopyrrolidin-2-ylidene]pyridine-2-carbohydrazide Crystal data

**Table:**

C_10_H_10_N_4_O_2_	*Z* = 2
*M**_r_* = 218.22	*F*(000) = 228
Triclinic, *P*1	*D*_x_ = 1.476 Mg m^−^^3^
*a* = 5.6332 (5) Å	Mo *K*α radiation, λ = 0.71073 Å
*b* = 7.6439 (7) Å	Cell parameters from 990 reflections
*c* = 11.7293 (11) Å	θ = 2.7–24.0°
α = 95.859 (8)°	µ = 0.11 mm^−^^1^
β = 96.353 (8)°	*T* = 293 K
γ = 99.682 (8)°	Plate, colourless
*V* = 491.04 (8) Å^3^	0.18 × 0.12 × 0.04 mm

### N'-[(E)-5-Oxopyrrolidin-2-ylidene]pyridine-2-carbohydrazide Data collection

**Table:**

Rigaku Xcalibur Eos diffractometer	1734 independent reflections
Radiation source: fine-focus sealed X-ray tube, Enhance (Mo) X-ray Source	1120 reflections with *I* > 2σ(*I*)
Graphite monochromator	*R*_int_ = 0.037
Detector resolution: 16.1593 pixels mm^-1^	θ_max_ = 25.0°, θ_min_ = 2.7°
ω scans	*h* = −6→6
Absorption correction: multi-scan (CrysAlis PRO; Rigaku OD, 2021)	*k* = −9→8
*T*_min_ = 0.716, *T*_max_ = 1.000	*l* = −13→13
4130 measured reflections	

### N'-[(E)-5-Oxopyrrolidin-2-ylidene]pyridine-2-carbohydrazide Refinement

**Table:**

Refinement on *F*^2^	Hydrogen site location: inferred from neighbouring sites
Least-squares matrix: full	H-atom parameters constrained
*R*[*F*^2^ > 2σ(*F*^2^)] = 0.049	*w* = 1/[σ^2^(*F*_o_^2^) + (0.0489*P*)^2^] where *P* = (*F*_o_^2^ + 2*F*_c_^2^)/3
*wR*(*F*^2^) = 0.121	(Δ/σ)_max_ < 0.001
*S* = 1.01	Δρ_max_ = 0.17 e Å^−^^3^
1734 reflections	Δρ_min_ = −0.17 e Å^−^^3^
146 parameters	Extinction correction: SHELXL2018 (Sheldrick, 2015*b*), Fc^*^=kFc[1+0.001xFc^2^λ^3^/sin(2θ)]^-1/4^
0 restraints	Extinction coefficient: 0.019 (4)
Primary atom site location: dual	

### N'-[(E)-5-Oxopyrrolidin-2-ylidene]pyridine-2-carbohydrazide Special details

**Table:**

Geometry. All esds (except the esd in the dihedral angle between two l.s. planes) are estimated using the full covariance matrix. The cell esds are taken into account individually in the estimation of esds in distances, angles and torsion angles; correlations between esds in cell parameters are only used when they are defined by crystal symmetry. An approximate (isotropic) treatment of cell esds is used for estimating esds involving l.s. planes.
Refinement. All H atoms were placed geometrically (C—H = 0.93–0.97 Å and N—H = 0.86 Å) and refined as riding atoms, with *U*_iso_(H) = 1.2*U*_eq_(carrier).

### N'-[(E)-5-Oxopyrrolidin-2-ylidene]pyridine-2-carbohydrazide Fractional atomic coordinates and isotropic or equivalent isotropic displacement parameters (Å^2^)

**Table:**

	*x*	*y*	*z*	*U*_iso_*/*U*_eq_	
O1	−0.2261 (3)	0.2881 (3)	0.25651 (14)	0.0656 (6)	
O2	0.6944 (3)	0.9414 (2)	0.63831 (14)	0.0578 (6)	
N1	0.1853 (4)	0.3516 (3)	0.05028 (15)	0.0402 (5)	
N2	0.1546 (4)	0.4460 (3)	0.26871 (15)	0.0416 (6)	
H2	0.278937	0.471361	0.233005	0.050*	
N3	0.1655 (4)	0.5201 (3)	0.38345 (15)	0.0392 (5)	
N4	0.4061 (3)	0.7230 (2)	0.52866 (14)	0.0372 (5)	
H4	0.308537	0.706286	0.579587	0.045*	
C1	0.2008 (5)	0.3059 (3)	−0.0616 (2)	0.0467 (7)	
H1	0.341810	0.352645	−0.090781	0.056*	
C2	0.0195 (5)	0.1935 (3)	−0.1359 (2)	0.0482 (7)	
H2A	0.039786	0.164612	−0.212720	0.058*	
C3	−0.1905 (5)	0.1248 (3)	−0.0955 (2)	0.0470 (7)	
H3	−0.315240	0.048043	−0.143784	0.056*	
C4	−0.2127 (5)	0.1729 (3)	0.01988 (19)	0.0422 (6)	
H4A	−0.354490	0.130957	0.049950	0.051*	
C5	−0.0224 (4)	0.2831 (3)	0.08845 (18)	0.0341 (6)	
C6	−0.0415 (4)	0.3380 (3)	0.21342 (19)	0.0380 (6)	
C7	0.3606 (4)	0.6329 (3)	0.41794 (18)	0.0320 (6)	
C8	0.5706 (4)	0.6949 (3)	0.35502 (19)	0.0410 (6)	
H8A	0.518851	0.753902	0.289671	0.049*	
H8B	0.643339	0.595005	0.327358	0.049*	
C9	0.7488 (4)	0.8250 (3)	0.44387 (19)	0.0456 (7)	
H9A	0.897665	0.779229	0.460945	0.055*	
H9B	0.788117	0.940108	0.415897	0.055*	
C10	0.6218 (4)	0.8415 (3)	0.5493 (2)	0.0401 (6)	

### N'-[(E)-5-Oxopyrrolidin-2-ylidene]pyridine-2-carbohydrazide Atomic displacement parameters (Å^2^)

**Table:**

	*U*^11^	*U*^22^	*U*^33^	*U*^12^	*U*^13^	*U*^23^
O1	0.0498 (12)	0.0928 (15)	0.0417 (11)	−0.0198 (11)	0.0219 (10)	−0.0139 (10)
O2	0.0616 (13)	0.0639 (13)	0.0365 (10)	−0.0063 (10)	0.0007 (9)	−0.0121 (9)
N1	0.0406 (13)	0.0460 (13)	0.0325 (11)	0.0000 (10)	0.0127 (10)	0.0017 (9)
N2	0.0420 (13)	0.0517 (13)	0.0267 (11)	−0.0043 (10)	0.0146 (10)	−0.0063 (9)
N3	0.0419 (13)	0.0470 (13)	0.0267 (11)	0.0031 (11)	0.0104 (10)	−0.0025 (9)
N4	0.0370 (12)	0.0481 (12)	0.0249 (10)	0.0038 (10)	0.0097 (9)	−0.0022 (9)
C1	0.0459 (16)	0.0586 (17)	0.0343 (15)	−0.0001 (14)	0.0166 (13)	0.0024 (12)
C2	0.0614 (19)	0.0546 (17)	0.0277 (13)	0.0075 (15)	0.0103 (13)	0.0003 (12)
C3	0.0514 (17)	0.0502 (16)	0.0336 (14)	0.0000 (13)	0.0004 (13)	−0.0014 (12)
C4	0.0383 (15)	0.0499 (15)	0.0340 (14)	−0.0036 (12)	0.0080 (11)	−0.0005 (11)
C5	0.0362 (14)	0.0373 (14)	0.0274 (12)	0.0026 (11)	0.0070 (11)	0.0018 (10)
C6	0.0393 (15)	0.0387 (14)	0.0336 (14)	−0.0013 (12)	0.0106 (12)	0.0003 (11)
C7	0.0353 (14)	0.0339 (13)	0.0279 (12)	0.0081 (11)	0.0073 (11)	0.0021 (10)
C8	0.0437 (15)	0.0448 (15)	0.0342 (14)	0.0054 (12)	0.0123 (12)	−0.0001 (11)
C9	0.0374 (15)	0.0535 (16)	0.0424 (15)	0.0006 (13)	0.0081 (12)	−0.0014 (12)
C10	0.0407 (16)	0.0423 (15)	0.0354 (14)	0.0064 (13)	0.0019 (12)	0.0005 (12)

### N'-[(E)-5-Oxopyrrolidin-2-ylidene]pyridine-2-carbohydrazide Geometric parameters (Å, º)

**Table:**

O1—C6	1.226 (2)	C2—C3	1.366 (3)
O2—C10	1.216 (3)	C3—H3	0.9300
N1—C1	1.340 (3)	C3—C4	1.390 (3)
N1—C5	1.342 (3)	C4—H4A	0.9300
N2—H2	0.8600	C4—C5	1.368 (3)
N2—N3	1.396 (2)	C5—C6	1.504 (3)
N2—C6	1.322 (3)	C7—C8	1.498 (3)
N3—C7	1.276 (3)	C8—H8A	0.9700
N4—H4	0.8600	C8—H8B	0.9700
N4—C7	1.382 (3)	C8—C9	1.518 (3)
N4—C10	1.370 (3)	C9—H9A	0.9700
C1—H1	0.9300	C9—H9B	0.9700
C1—C2	1.376 (3)	C9—C10	1.500 (3)
C2—H2A	0.9300		
			
C1—N1—C5	116.3 (2)	C4—C5—C6	120.1 (2)
N3—N2—H2	119.1	O1—C6—N2	124.8 (2)
C6—N2—H2	119.1	O1—C6—C5	121.7 (2)
C6—N2—N3	121.87 (19)	N2—C6—C5	113.48 (19)
C7—N3—N2	112.39 (18)	N3—C7—N4	121.6 (2)
C7—N4—H4	123.3	N3—C7—C8	130.20 (19)
C10—N4—H4	123.3	N4—C7—C8	108.2 (2)
C10—N4—C7	113.35 (19)	C7—C8—H8A	110.8
N1—C1—H1	118.2	C7—C8—H8B	110.8
N1—C1—C2	123.6 (2)	C7—C8—C9	104.83 (17)
C2—C1—H1	118.2	H8A—C8—H8B	108.9
C1—C2—H2A	120.4	C9—C8—H8A	110.8
C3—C2—C1	119.3 (2)	C9—C8—H8B	110.8
C3—C2—H2A	120.4	C8—C9—H9A	110.7
C2—C3—H3	120.9	C8—C9—H9B	110.7
C2—C3—C4	118.2 (2)	H9A—C9—H9B	108.8
C4—C3—H3	120.9	C10—C9—C8	105.28 (18)
C3—C4—H4A	120.5	C10—C9—H9A	110.7
C5—C4—C3	118.9 (2)	C10—C9—H9B	110.7
C5—C4—H4A	120.5	O2—C10—N4	125.0 (2)
N1—C5—C4	123.7 (2)	O2—C10—C9	126.9 (2)
N1—C5—C6	116.2 (2)	N4—C10—C9	108.08 (19)
			
N1—C1—C2—C3	0.8 (4)	C3—C4—C5—N1	1.5 (4)
N1—C5—C6—O1	177.6 (2)	C3—C4—C5—C6	179.7 (2)
N1—C5—C6—N2	−0.9 (3)	C4—C5—C6—O1	−0.8 (4)
N2—N3—C7—N4	179.33 (18)	C4—C5—C6—N2	−179.3 (2)
N2—N3—C7—C8	0.2 (3)	C5—N1—C1—C2	−0.8 (4)
N3—N2—C6—O1	−2.4 (4)	C6—N2—N3—C7	−174.0 (2)
N3—N2—C6—C5	176.01 (18)	C7—N4—C10—O2	176.4 (2)
N3—C7—C8—C9	−178.2 (2)	C7—N4—C10—C9	−3.8 (3)
N4—C7—C8—C9	2.6 (2)	C7—C8—C9—C10	−4.6 (2)
C1—N1—C5—C4	−0.3 (3)	C8—C9—C10—O2	−175.0 (2)
C1—N1—C5—C6	−178.6 (2)	C8—C9—C10—N4	5.2 (3)
C1—C2—C3—C4	0.4 (4)	C10—N4—C7—N3	−178.6 (2)
C2—C3—C4—C5	−1.5 (4)	C10—N4—C7—C8	0.7 (3)

### N'-[(E)-5-Oxopyrrolidin-2-ylidene]pyridine-2-carbohydrazide Hydrogen-bond geometry (Å, º)

**Table:**

*D*—H···*A*	*D*—H	H···*A*	*D*···*A*	*D*—H···*A*
N4—H4···O1^i^	0.86	2.02	2.822 (2)	154

Symmetry code: (i) −*x*, −*y*+1, −*z*+1.
