# Supplementary material for: N′-[(E)-5-Oxopyrrolidin-2-yl­idene]pyridine-2-carbohydrazide
Source: IUCrdata. 2025 Oct 28;10(Pt 10):x250896. doi: 10.1107/S241431462500896X (PMC12598890; doi:10.1107/S241431462500896X)
Supplement: Supplementary file 3 [file x-10-x250896-sup3.pdf]

Electronic Supporting Information

Synthesis and crystal structure of N'-[(2E)-5-oxopyrrolidin-2-yl-idene]pyridine-2-carbohydrazide

Yurii S. Bibik,[a]* Hanna V. Ivanova,[a] Oleksandr V. Vaschenko,[a] Dmytro M. Khomenko,[ab] Roman O. Doroshchuk,a[bc] Ilona V. Raspertova,[ab] Alexandru-Constantin Stoica[d] and Rostyslav D. Lampeka[a]

[a]Department of Chemistry, Kyiv National Taras Shevchenko University, Hetmana, Pavla Skoropadskogo, st. 12, Kyiv, Ukraine,

[b]Enamine Ltd., Winston Churchil, st. 78, Kyiv 02094, Ukraine,

[c]ChemBioCenter, Kyiv National Taras Shevchenko University, Hetman Pavlo, Skoropadskyi Street 12, Kyiv, 01601, Ukraine,

[d]Petru Poni" Institute of Macromolecular Chemistry, Aleea Gr. Ghica, Voda, 41A, 700487 Iaşi, Romania

Correspondence email: yurii.bibik@knu.ua

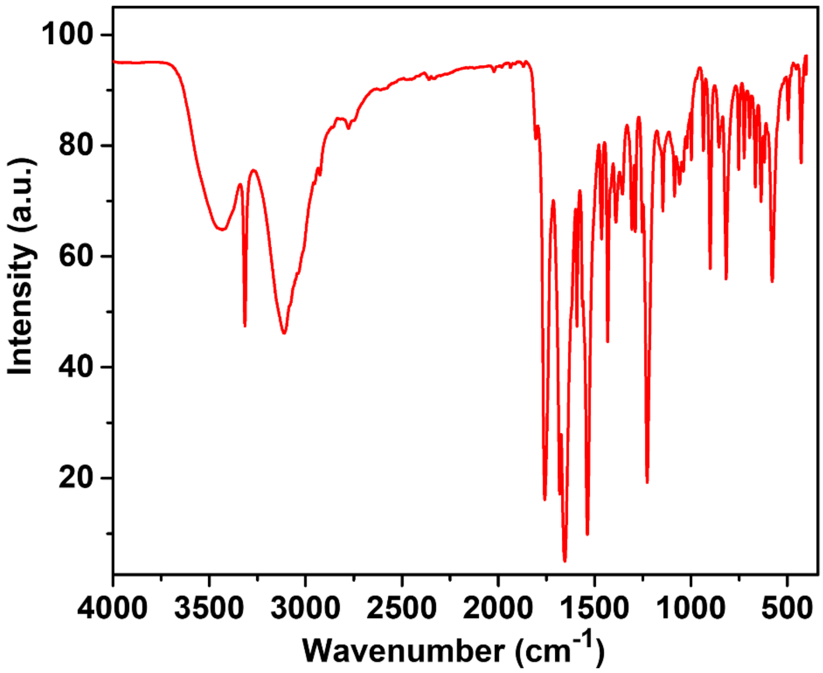

Figure S1. IR spectrum of the title compound in KBr.

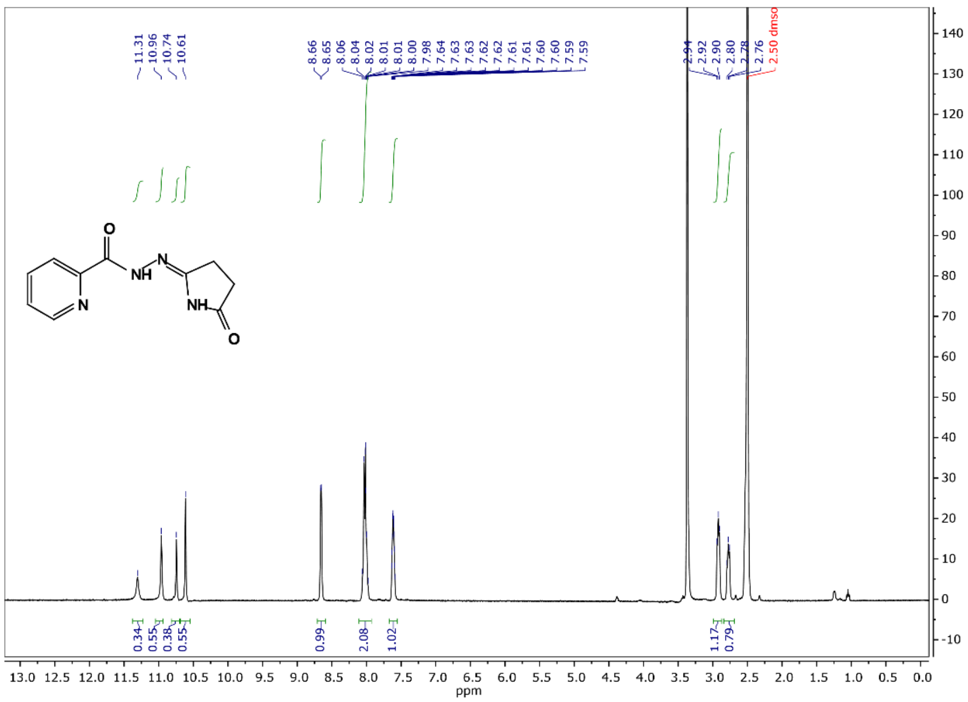

Figure S2. ^{1}H NMR spectrum of the title compound in dmso-d_6.
